# Implantation of Sutureless Scleral-Fixated Carlevale Intraocular Lens (IOL) in Patients with Insufficient Capsular Bag Support: A Retrospective Analysis of 100 Cases at a Single Center †

**DOI:** 10.3390/jcm14124378

**Published:** 2025-06-19

**Authors:** Jan Strathmann, Sami Dalbah, Tobias Kiefer, Nikolaos E. Bechrakis, Theodora Tsimpaki, Miltiadis Fiorentzis

**Affiliations:** Department of Ophthalmology, Eye Cancer Center Essen, University Hospital Essen, Hufelandstr. 55, 45147 Essen, Germany

**Keywords:** Carlevale IOL, scleral fixation, secondary lens implant, aphakia

## Abstract

**Background/Objectives**: Different surgical techniques are available in cases of missing or insufficient capsular bag support. Next to the anterior chamber or iris-fixated intraocular lenses (IOL), the implantation of the Carlevale IOL provides a sutureless and scleral fixated treatment method. **Methods**: In a retrospective single-center study, the perioperative data of 100 patients who consecutively received a scleral fixated Carlevale IOL combined with a 25 gauge (G) pars plana vitrectomy between September 2021 and June 2024 were investigated. The intraoperative and postoperative results were analyzed in terms of complication rates and refractive outcomes. **Results**: IOL dislocation was the most common surgical indication (50%) for sutureless Carlevale IOL implantation, followed by postoperative aphakia in 35 patients (35%). Nearly every fourth patient (24%) had a preoperative traumatic event, and 21% had pseudoexfoliation (PEX) syndrome. The average surgery time was 60.2 (±20.1) min. Intraoperative intraocular hemorrhage occurred in seven cases, and IOL haptic breakage in two patients. Temporary intraocular pressure fluctuations represented the most common postoperative complications (28%). Severe complications such as endophthalmitis or retinal detachment were not observed in our cohort. The mean refractive prediction error was determined in 67 patients and amounted to an average of −0.7 ± 2.0 diopters. The best corrected visual acuity (BCVA) at the last postoperative follow-up showed an improvement of 0.2 ± 0.5 logMAR (*n* = 76) compared to the preoperative BCVA (*p* = 0.0002). The postoperative examination was performed in 72% of the patients, and the mean follow-up period amounted to 7.2 ± 6.4 months. **Conclusions**: Overall, sutureless and scleral fixated implantation of the Carlevale IOL represents a valuable therapeutic option in the treatment of aphakia and lens as well as IOL dislocation in the absence of capsular bag support with minor postoperative complications and positive refractive outcomes.

## 1. Introduction

Aphakia and insufficient capsular bag support may have different causes [1,2,3]. Typical risk factors are pseudoexfoliation (PEX) syndrome, history of trauma, systemic connective tissue diseases such as Marfan syndrome, complicated cataract surgery, and previous ocular surgery in general, especially pars plana vitrectomy [4,5]. Lensectomy in patients with infantile cataract is another cause of temporary aphakia [6]. As these risk factors are not uncommon and can occur simultaneously, complicated aphakia or insufficient capsular bag support with lens luxation, accompanied by serious vision impairment, require appropriate treatment strategies.

Regarding secondary intraocular lens (IOL) implantation, various surgical techniques have been published and explored. The exact surgical approach depends on the lens status and surrounding anatomical structures. The most important consideration is whether adequate capsular bag support is available. If the lens capsule is still intact, the IOL can be repositioned, or a secondary IOL can be implanted in the sulcus. In cases of complete capsular bag insufficiency, a secondary IOL can be placed either into the anterior chamber angle (AC-IOL), enclavated in the iris (IF-IOL), or fixed to the sclera [7]. AC- and IF-IOL are quick and easy to implant with minimal surgical effort and surgery time but have a certain potential for complications such as secondary glaucoma or pupil distortion and increased surgery-induced astigmatism due to the anatomical proximity to the cornea and a large access incision [8,9]. Additionally, IF-IOL should not be the first choice for patients with a positive history of iris trauma or iritis. SF-IOL, on the other hand, are technically more demanding but guarantee an anatomically correct lens position and intraocular architecture [9,10].

There are different approaches to scleral IOL fixation, including sutured and sutureless procedures [11,12]. The Yamane technique presents an established method for sutureless intrascleral fixation of a posterior chamber intraocular lens, in which the haptics of a three-piece IOL are externalized through the ciliary sulcus, cauterized at the end of the haptics to create a flange and then fixed in a lamellar scleral tunnel [13].

Sutured techniques or techniques such as the Yamane technique carry the risk of decentration and instability owing to breakage of the suture material or inappropriate positioning of the created flange. However, these surgeries have a short intervention duration and rapid visual recovery [14,15]. Despite the multitude of options, there is currently no gold standard for IOL implantation in the absence of capsular bag support or aphakia [16].

The Carlevale IOL is the sole on-label solution available in Europe, as it is a lens specifically manufactured and approved for sutureless scleral fixation by the European Medicines Agency (EMA). However, it has not yet received approval from the U.S. Food and Drug Administration (FDA) [17,18]. To date, satisfactory results have been reported in terms of refractive outcome and complication rates. Furthermore, various implantation techniques and modifications, including subconjunctival positioning of the anchors and the creation of scleral pockets or flaps for instrascleral fixation, are already available [19,20].

In this study, we present a retrospective analysis of patients following scleral fixated implantation of the Carlevale IOL by performing scleral pockets. In particular, surgical indications, refractive outcomes, and complication rates were investigated and reported. Additionally, refractive outcomes and complication rates were compared between our standard technique, which includes a Carlevale implantation combined with a vitrectomy, and interventions that include accompanying procedures such as vitreoretinal surgery or keratoplasty.

## 2. Materials and Methods

### 2.1. Study Design, Patients, and Data Collection

In this retrospective monocentric study, the perioperative data of patients with insufficient capsular bag support or the presence of aphakia who were provided with implantation of a Carlevale IOL at the University Eye Clinic in Essen between 09/2021 and 06/2024 were analyzed. All available health records, including operative reports as well as physician letters, of 100 consecutive patients were studied to collect preoperative (patient demographics and ophthalmologic history), operative, and postoperative data. Pre- and postoperative eye status were assessed, including best-corrected visual acuity (BCVA), if refractive assessment was available, Goldmann applanation tonometry, slit-lamp examination of the anterior segment, and fundoscopy in mydriasis. Biometry was performed using the IOL Master 500 (Carl Zeiss Meditec AG, Jena, Germany) optical biometry, and IOL power was calculated depending on the axial length of the eye using appropriate formulas (SRK/T, Haigis, Hoffer Q). Follow-up information could only be obtained for patients who underwent postoperative follow-up examinations at our institution. The first postoperative refractive check-up was performed after six to eight weeks. The study was performed in accordance with the Declaration of Helsinki and was approved by the Ethics Committee of the University Duisburg-Essen.

### 2.2. Lens Design

**Figure 1:**
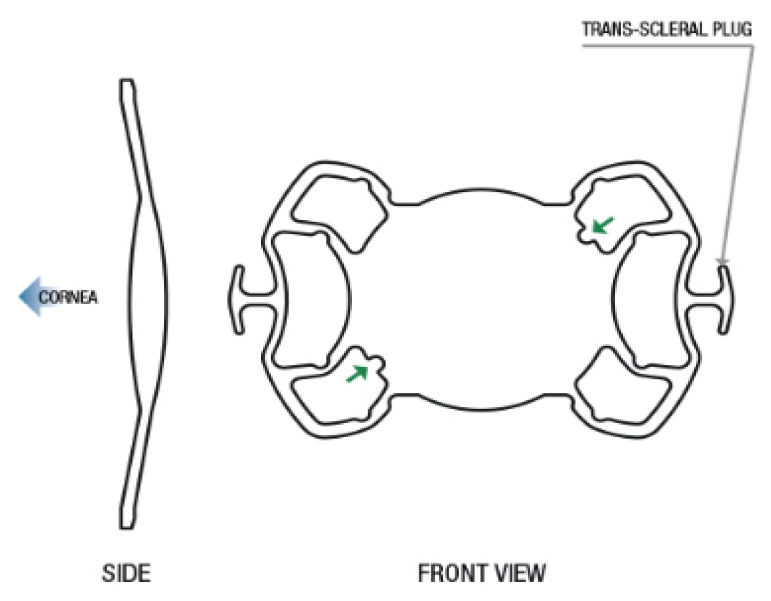
Morphology of Carlevale FIL-SSF IOL. Green arrows are pointing on special markings for correct intraoperative alignment [21].

The Carlevale IOL (Carlevale FIL-SSF IOL; Soleko, Pontecorvo, Italy) is a lens especially approved for sutureless scleral fixation. It is a one-piece hydrophilic acrylic lens with a large optic with a diameter of 6.5 mm and an overall length of 13.2 mm. This lens is hypothesized to be particularly stable because of two T-plugs used for fixation in the scleral pockets. Additionally, the lens is equipped with four so-called “ears” which are positioned in the sulcus region of the eye and shall provide supplementary stability. To protect the anterior segment of the eye, the IOL is angled posteriorly by 10 degrees. For correct intraoperative alignment, the lens has two special markings (Figure 1).

### 2.3. Surgical Technique

**Figure 2:**
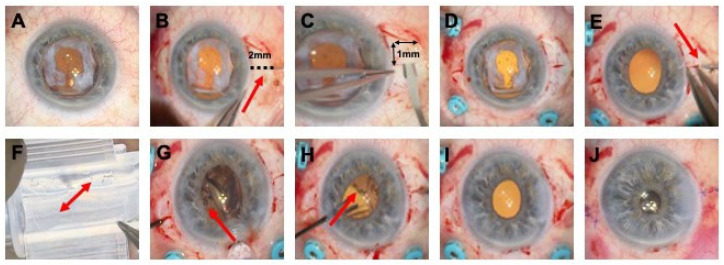
Carlevale IOL implantation in case of IOL dislocation. Initial finding with a loose IOL resting on the anterior vitreous body (**A**). Following the conjunctival opening, two meridional partial thickness incisions 2 mm away from the limbus are prepared (**B**). Scleral pockets (1 mm on each side) are prepared (**C**). Standard setting in our clinic with 25G pars plana vitrectomy (**D**). After removal of the IOL and complete vitrectomy, scleral incisions are opened 1–1.5 mm away from the limbus with a 25G lance (**E**). Positioning the lens in the shooter in the correct alignment as it should be in the eye. Note the marking spots that indicate the 10-degree angle of the lens (red arrows) (**F**). Lens implantation and holding of the leading anchor before externalization (red arrow) (**G**). Following, “hand-shake” technique externalization of the second anchor is performed (**H**). Note the marking spot indicating the correct alignment of the lens (red arrow) (**H**). Scleral pockets closure with 10-0 nylon suture (**I**). End of the surgery following conjunctival closure (**J**).

The surgical procedure included lens or IOL explantation and implantation of a scleral fixated aspheric Carlevale intraocular lens. In our cohort, no toric IOL was implanted. The surgery was always combined with a 25G complete pars plana vitrectomy (standard procedure), and depending on other pathologies requiring surgery, appropriate concomitant procedures were performed as well (combination procedure). The surgeries were conducted by three different surgeons, mostly under general anesthesia and, in one case, under locoregional anesthesia. After the initial conjunctival opening, two opposite meridional incisions were made to dissect the scleral pockets. These were made 180° apart, predominantly in the 3 and 9 o’clock positions, and each 2.0 mm away from the limbus. A sclerocorneal tunnel with a diameter of 2.2 to 3.0 mm was then prepared, mostly at the 12 o’clock position. Three 25G trocars were used for a pars plana vitrectomy. After vitrectomy, the subluxated or luxated lens or IOL was explanted. Next, the Carlevale IOL was inserted into the anterior chamber via the tunnel using a shooter. Through sclerotomies in the middle of the scleral pockets, the haptics were externalized and placed in the scleral pockets using 25G forceps. In most cases, air or balance salt solution (BSS) was used as intraocular tamponade; in rarer cases, also gas (SF6) or silicone oil. Finally, the meridional incisions were sutured with 10-0 nylon, the sclerotomies with 7-0 PDS, and the conjunctiva with 7-0 vicryl (Figure 2, Video S1).

### 2.4. Statistical Analysis

Descriptive statistics were computed by using Microsoft Excel 2022 (Microsoft^®^ Excel). For the analysis, BCVA values were converted from decimal to logarithm of the minimum angle of resolution (logMAR). Continuous variables are expressed as mean ± standard deviation (SD), and categorical variables are expressed as absolute numbers with corresponding percentages. Refractive prediction error was defined as the deviation of the postoperative spherical equivalent (SE) from the preoperatively defined target refraction. *p*-values were calculated using paired *t*-tests for continuous variables, chi-square tests for categorical variables, and one-way analyses of variance (ANOVA) for comparing the means of more than two groups. GraphPad Prism version 10.2.3 (©2024 GraphPad Software; Boston, MA, USA) was utilized for these analyses. Statistical significance was set at *p* < 0.05.

## 3. Results

### 3.1. Patient Demographics and Clinical Characteristics

The average patient age was 62.8 ± 22.5 years, with the youngest patient being 4.9 and the oldest patient being 91.9 years old. Ophthalmologic history revealed prior ophthalmic surgery in 94% of the cohort eyes. Vitrectomy was previously performed in 50% of the patients. Typical risk factors for lens dislocation were present, with a positive trauma history in approximately one-fourth (24%) of the patients and the presence of pseudoexfoliation syndrome in 21%. Postoperative aphakia following lensectomy for congenital cataracts was present in 8% and Marfan syndrome in 2% of the patients.

**Figure 3:**
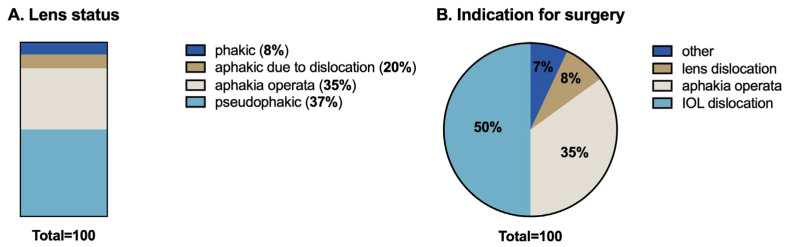
(**A**) Lens status; (**B**) indication for surgery. Other indications include IOL opacity, primary zonular insufficiency, and anterior chamber-associated complications.

At the time of surgery, 55% of patients were aphakic. Aphakia was attributed to dislocation in 20 patients, whereas 37% were pseudophakic and 8% of the patients showed a dislocated lens. Regarding the indications for surgery, IOL subluxations or complete dislocations accounted for the majority of the cases (50%), followed by aphakia operata (35%) and lens dislocation (8%) (Figure 3).

Rare indications were IOL opacities in three cases: primary zonular insufficiency (2%) or anterior chamber-associated complications, such as iris shaving (1%) or corneal decompensation (1%).

The average axial length was 24.6 ± 2.1 mm, and the preoperative BCVA was 0.9 ± 0.7 logMAR. Detailed refractive baseline data are shown in Table 1.

### 3.2. Operative Data

A 25G pars plana vitrectomy was performed in all the patients (100%), and air was the most commonly used intraocular tamponade (83%). Less frequently used tamponades were BSS (11%), sulfur hexafluoride gas (SF6) (5%) and silicone oil (1%).

In approximately every fourth operation (27%), an accompanying procedure was performed in conjunction with the standard procedure of Carlevale implantation combined with a vitrectomy. These procedures are summarized in Table 2.

The mean procedure time was 60.2 ± 20.1 min. Intraoperative complications occurred in 12% of the cases, with intraocular hemorrhage being the most frequent one (7/100). An overview of all intraoperative complications is given in Table 3.

Retinal tears occurred more frequently in combination procedures (7.4 versus 0.0%; *p* = 0.02), while there were no differences regarding other intraoperative complications between standard and combination procedures.

### 3.3. Postoperative Data

Postoperative follow-up was realized in 72% of the patients, with an average follow-up time of 7.2 (0–26) months.

**Figure 4:**
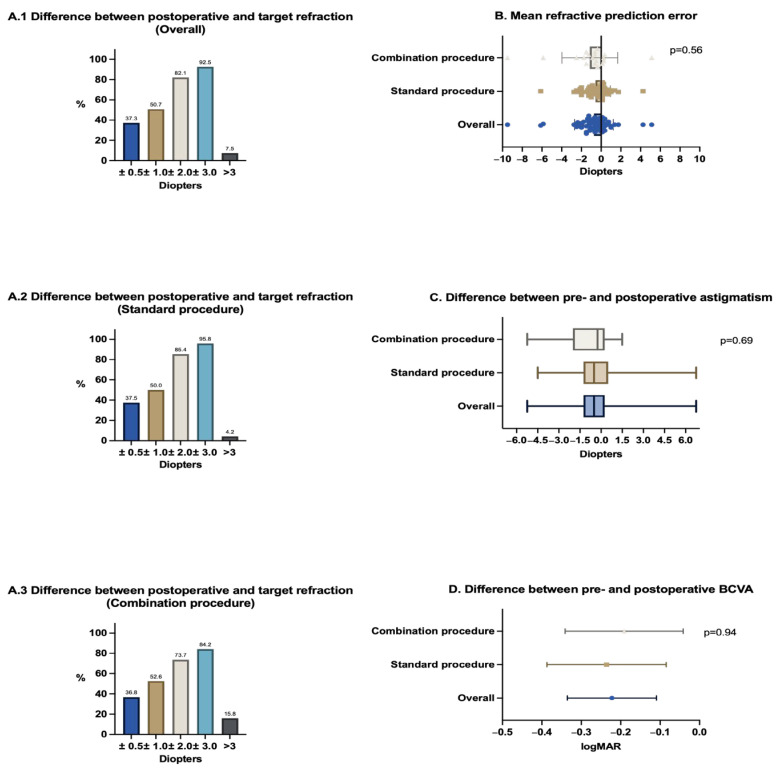
Refractive results. (**A.1**–**A.3**): Difference between postoperative and target refraction. Columns indicate the percentage of eyes that achieved refractive results in the corresponding target areas. (**A.1**) refers to the overall, (**A.2**) to the standard procedure group, and (**A.3**) to the combination procedure group. (**B**): Mean refractive prediction error. Scatter plot presenting data as mean with standard deviation. (**C**): Difference between pre- and postoperative astigmatism. Box plot: min to max. (**D**): Difference between pre- and postoperative BCVA. Column mean, error bars: mean with 95% confidence interval. Mean refractive prediction error = difference between postoperative spherical equivalent and target refraction. *p*-values were calculated using a one-way analysis of variance (ANOVA).

In the overall group, the average postoperative BCVA measured at the last follow-up was 0.6 ± 0.6 logMAR (*n* = 76), showing an improvement of 0.2 ± 0.5 logMAR compared to the preoperative BCVA (*p* = 0.0002). The difference between the average pre- and postoperative astigmatism was −0.6 ± 1.8 dpt. (*n* = 54; *p* = 0.02). The mean refractive prediction error, defined as the difference between the postoperative spherical equivalent and the target refraction, amounted to −0.7 ± 2.0 dpt. (*n* = 67). Detailed refractive results are shown in Figure 4.

In the overall group, 37.3% of the eyes achieved a postoperative refraction within ±0.5 dpt., while half of the eyes (50.7%) achieved it within ±1.0 dpt. of the target refraction. Similar proportions are observed in the subgroups: in the standard procedure group, 37.5% of the eyes were within ± 0.5 dpt. and 50.0% within ±1.0 dpt., while in the combination procedure group, 36.8% were within ±0.5 dpt. and 52.6% within ±1.0 dpt. of the target refraction (Figure 4A.1–A.3).

Regarding the mean refractive prediction error (ranging from −0.57 ± 0.22 to −1.14 ± 0.65; *p* = 0.56) and the differences between pre- and postoperative astigmatism (ranging from −0.44 ± 0.28 to −0.88 ± 0.43; *p* = 0.69), as well as between pre- and postoperative BCVA (ranging from −0.19 ± 0.07 to −0.24 ± 0.08; *p* = 0.94), no statistically significant differences were observed between the overall, standard and combination procedure groups (Figure 4B–D).

Among the postoperative complications, fluctuations in intraocular pressure were observed in almost one-third of patients. Hypo- (13%) and hypertension (15%) were equally common. Out of the patients with postoperative hypertension, six patients had pre-existing glaucoma. In eight patients, local anti-glaucomatous therapy was sufficient, while seven patients required additional systemic anti-glaucomatous therapy. Glaucoma surgery was not necessary for any patient. To manage hypotension, topical corticosteroids were employed. Leakage through the sclerotomies was not observed, and hypotension regulated itself in all patients during their hospital stay.

Intraocular hemorrhages, both in the anterior chamber and vitreous cavity, were present in 14% of the patients postoperatively. All of these hemorrhages could be treated conservatively.

Postoperative macular edema occurred in five patients, which regressed under local therapy with topical NSAID or corticosteroids. Wound dehiscence, either of the conjunctiva or sclera, occurred in four patients and required surgical revision in three of these four cases.

The proportion of patients with wound dehiscence was statistically significantly higher in the combined procedure than in the standard procedure group (11.1 versus 1.4%; *p* = 0.03) (Table 4).

Serious complications, such as retinal detachment or endophthalmitis, occurred in none of the cases.

## 4. Discussion

Our study demonstrates the results following implantation of the sutureless scleral fixated Carlevale IOL with regard to visual rehabilitation, stability, and safety. With respect to postoperative complications and refraction, positive outcomes were observed in both cases with Carlevale implantation combined with vitrectomy and in patients where further surgical procedures were performed.

The average age of our cohort was 62.8 years, which is consistent with the literature and the peak age of onset of lens dislocations and aphakia [4,5]. The typical risk factors for lens dislocations, such as pseudoexfoliation syndrome, previous surgery, especially vitrectomy, and a positive trauma history [4], were also evident to a large amount in our patient cohort.

In 2018, Vounotrypidis et al. reported that previous pars plana vitrectomy was a major underreported risk factor in eyes that underwent secondary IOL implantation [5]. In accordance with this finding, 50% of the patients had a positive history of a previously conducted vitrectomy. In our cohort, the most common indications for previous vitrectomy were retinal detachment or penetrating globe injury. Complicated cataract surgery requiring anterior vitrectomy was present in only four patients.

Approximately one in four of our patients had a positive history of trauma, either penetrating globe injury (18%) or blunt ocular trauma (6%), whereas 4–17% have been documented in other studies focusing on secondary IOL implantation [5,17,22].

Giannakopoulos et al., Vounotrypidis et al., and Danese et al. report IOL dislocation as the most common surgical indication with 54.2% [17], 75.0% [5] and 71.4% [20] accordingly. In agreement with the literature, IOL dislocation was the most frequent indication for sutureless implantation of the Carlevale IOL in the present study.

In our institution, we performed a standard 25G pars plana vitrectomy to frequently remove the previous IOL or lens, to minimize vitreous-related complications, to facilitate the intraoperative management of the Carlevale IOL, and to secure access in case of a potential fall of the IOL into the vitreous cavity. Surgical methods, with and without combined vitrectomy, have been described in the literature. According to Fiore et al., who performed vitrectomy only when necessary, no differences were found between Carlevale implantation with and without combined vitrectomy, claiming that implantation of a Carlevale IOL can be feasible for anterior and posterior segment surgeons [23]. We believe that a complete vitrectomy should be performed, especially in cases where sub-/ or luxated IOL removal is needed and in cases with pseudoexfoliation syndrome, since insufficient mydriasis may lead to further complications if the first anchor of the lens cannot be grasped and externalized in the area of the anterior vitreous. In these cases, we prefer to let the lens fall into the posterior segment and proceed with further implantation maneuvers. Therefore, a complete vitrectomy is necessary. After performing a complete vitrectomy, we predominantly used air as intraocular tamponade, favoring it over BSS primarily due to its superior stability at the site of the vitrectomy ports. In the event of other pathologies, such as retinal breaks or retinal detachment, gas, and silicone oil were used.

The average operation time of 60 min was consistent with the experience of other working groups on scleral fixated IOL implantation [24]. Compared to iris-fixated IOL, such as the Artisan lens, which has an average intervention time of approximately 40 min, scleral fixated implantation techniques are associated with longer operation times owing to a more demanding surgical procedure [22,25].

Stable improvement in visual acuity of 0.2 logMAR was observed in our overall group following the implantation of the Carlevale IOL. Other groups reported an improvement of 0.3 to even 0.5 logMAR [17,24,26]. The average astigmatism change due to surgery amounted to −0.6 dpt. in our group and is comparable to values reported by Danese et al., who reported a mean surgically induced astigmatism of −0.8 dpt. [20]. The mean refractive prediction error, defined as the difference between the postoperative spherical equivalent and target refraction, was −0.7 dpt. in our institution and is higher than the values of −0.27 and −0.24 dpt. in other studies [26,27].

The poorer visual acuity improvements and the slightly larger mean refractive prediction error in our cohort compared to other published data could be due to the higher proportion of patients with a history of trauma—24% with a positive trauma history in our group compared to 4.1% in a collective published by Giannopoulos et al. [17]—or previous surgical procedures, since such eyes usually show a limited visual prognosis. In addition, precise IOL calculation may be more challenging in eyes with trauma.

Third-generation formulas were used for IOL calculation in our clinic. The use of newer formulas, such as the Barrett formula, which, according to a systematic review, has shown the smallest mean absolute error in several studies and thus appears to be more precise [28], could also lead to a lower mean refractive prediction error when using the Carlevale IOL.

In a systematic review of the implantation of a Carlevale IOL, Barbieri et al. summarized that intraoperative complications are rather rare and predominantly involve damage to the lens and intraocular bleeding [10]. This is supported by the findings of Raimondi et al. and Bontemps et al., who reported single cases of haptic rupture in 2 and 1.3% of the cases, respectively [19,22]. In accordance with these results, we observed intraoperative bleeding in 7% and haptic rupture in only 2% of patients.

Postoperative complications include intraocular pressure fluctuations, transient macular edema, and bleeding events [10,17,26,29,30]. Overall, the frequency of these postoperative complications was similar between our group and the literature. Only the incidence of postoperative intraocular bleeding was slightly higher in our cohort, with 14% compared to values of 3.1 to 4.7% for vitreous hemorrhage, according to Raimondi et al. [19]. The documented increased incidence, similar to the refractive results, could be attributed to the increased number of eyes with trauma in our study cohort and a potentially increased risk of bleeding. Another potential factor might be the anticoagulation status of the patients, which, unfortunately, was not investigated in our study. Additionally, in contrast to Raimondi et al. [19], we not only documented vitreous hemorrhages but also hemorrhages in the anterior segment of the eye. The performance of complete vitrectomy in all included patients could additionally contribute to a higher incidence of postoperative intraocular hemorrhage. However, it should be noted that all bleeding events in our cohort were self-limiting, did not require surgical revision, and had no relevance to visual acuity results in the long term.

The research group led by Schranz et al. described the occurrence of a reverse pupillary block in 30% of cases during scleral-fixated intraocular lens procedures [31]. Although this complication was not observed in our cases, it is a condition that should not be underestimated as it can cause severe problems like iris chafing, leading to pigment dispersion or uveitis glaucoma hyphema syndrome over time [31].

Carlevale IOL implantation could be performed as a stand-alone procedure but also in combination with accompanying measures such as vitreoretinal surgery, keratoplasty, or anterior chamber revisions, often necessary after trauma, which demonstrates a versatile range of applications.

Upon analyzing the refractive results of the overall, standard, and combination procedure groups, we observe comparable results without statistically significant differences. With regard to complications, it should be mentioned that retinal tears (7.4 versus 0%; *p* = 0.02) occurred more frequently intraoperatively and wound dehiscences (11.1 versus 1.4%; *p* = 0.03) more frequently postoperatively in the group with concomitant measures. Nevertheless, these complications could be managed successfully, and serious complications, such as retinal detachment or endophthalmitis, were not observed in any of the groups.

The use of hydrophilic lens materials and intraocular gas tamponades has long been known to increase the risk of IOL calcification [32]. We combined Carlevale IOL implantation with DMEK, as well as vitreoretinal surgery, using intraocular gas and silicone oil tamponade. Stable results can be reported in a short follow-up period following the procedure. However, the course and the occurrence of potential calcifications should be monitored. In addition, hydrophobic variants, such as Carlevale IOL with a hydrophobic coating, should also be taken into consideration but were not used in this patient group.

When using the Carlevale IOL, different implantation techniques are described, which ultimately depend on the surgeon’s skill and preference.

Fiore et al. compared two different scleral fixation methods with the creation of scleral pockets or flaps and demonstrated equally good results [23]. These findings were confirmed by Marolo et al., who reported stable refractive results and equal long-term safety, especially with regard to the occurrence of haptic extrusion over a minimum period of 24 months [33].

Danese et al. described the subconjunctival positioning of Carlevale IOL anchors [20]. Although this method causes less surgical trauma, a potentially increased risk of outward dislocation of the anchors with an accompanying high probability of endophthalmitis must be considered.

Certain limitations of our study must be acknowledged. The retrospective nature of the present analysis with partially incomplete data collection has to be taken into consideration. Another point is the partial lack of refractive data at baseline and postoperatively due to inpatient admission in an emergency setting, with a lack of precise refractive diagnostics and partly undocumented refractive data in the included patient cohort.

Further points that were not investigated in our study were endothelial cell loss caused by the operation, the frequency of postoperative corneal edema, or the incidence of reverse pupillary block.

An advantage of Carlevale IOL implantation compared to sutured scleral fixated IOL implantation and, e.g., the Yamane technique, is greater stability and less IOL tilting [26]. Unfortunately, we did not assess the extent of tilting or stability of the lens; however, our patients did not report any such complaints.

## 5. Conclusions

Sutureless and scleral fixated implantation of the Carlevale IOL plays an important role as the currently only on-label procedure for the treatment of aphakia and lens or IOL dislocation in the absence of capsular bag support. In our cohort, satisfactory visual outcomes, a low-risk profile, and a wide range of applications were demonstrated for Carlevale IOL implantation, including combination procedures.

Nevertheless, the selection of the correct lens and implantation technique in general, as well as in cases of capsular bag insufficiency, depends on patient-specific factors and the surgeon’s experience. Future studies with extended follow-up periods should prioritize the assessment of long-term visual acuity outcomes and the potential development of long-term complications.

## Figures and Tables

**Table 1:** Refractive baseline data.

Refractive Baseline Data	Overall (*n* = 100)
Axial length	24.6 ± 2.1
Preoperative refraction (dpt.)	7.2 ± 6.3 (*n* = 78)
Preoperative astigmatism (dpt).	−1.7 ± 1.5 (*n* = 72)
Preoperative spherical equivalent (dpt.)	5.9 ± 6.5 (*n* = 72)
Preoperative BCVA (logMAR)	0.9 ± 0.7
Target refraction (dpt.)	−0.8 ± 1.1
IOL calculation (dpt.)	19.3 ± 5.3

Data presented as average ± standard deviation. Dpt.: diopters; BCVA: best corrected visual acuity; IOL: intraocular lens.

**Table 2:** Accompanying procedures.

Accompanying Procedures	Pathology	Overall
ILM peeling	Epiretinal membrane	8/100
Endolaser photocoagulation	Retinal defect	10/100
Anterior chamber procedures *	Iris defect (mostly traumatic)	8/100
Keratoplasty ^+^	Corneal decompensation	4/100

Data presented as relative frequencies. ILM: internal limiting membrane; * iris suture, iridoplasty, iridectomy, anterior chamber revision; ^+^ perforating or descemet membrane endothelial keratoplasty.

**Table 3:** Intraoperative complications.

Intraoperative Complications	Overall (*n* = 100)	Standard Procedure ^+^ (*n* = 73)	Combination Procedure * (*n* = 27)	*p*-Value
Intraocular hemorrhage	7.0	6.8	7.4	0.92
Haptic breakage	2.0	2.7	0.0	0.39
Retinal tears	2.0	0.0	7.4	0.02
Sphincter tear	1.0	1.4	0.0	0.54

Data presented as percentages. *p*-values related to the comparison of standard and combination procedures. ^+^ Carlevale implantation plus vitrectomy. * ILM peeling, endolaser photocoagulation, anterior chamber procedures, or keratoplasty.

**Table 4:** Postoperative complications.

Postoperative Complications	Overall (*n* = 100)	Standard Procedure ^+^ (*n* = 73)	Combination Procedure * (*n* = 27)	*p*-Value
Hypertension (≥24 mmHg)	15.0	12.3	22.2	0.22
Hypotension (≤6 mmHg)	13.0	13.7	11.1	0.73
Intraocular hemorrhage	14.0	15.1	11.1	0.61
CME	5.0	6.8	0.0	0.16
Wound dehiscence	4.0	1.4	11.1	0.03

Data presented as percentages. *p*-values related to the comparison of standard and combination procedure. CME: cystoid macular edema. ^+^ Carlevale implantation plus vitrectomy. * ILM peeling, endolaser photocoagulation, anterior chamber procedures, or keratoplasty.

## Data Availability

All data generated or analyzed during this study are included in this. Further inquiries can be directed to the corresponding author.

## Supplementary Materials

The following supporting information can be downloaded at: https://www.mdpi.com/article/10.3390/jcm14124378/s1, Video S1: Implantation of Carlevale IOL.
